# Unexpected silicon localization in calcium carbonate exoskeleton of cultured and fossil coccolithophores

**DOI:** 10.1038/s41598-023-34003-3

**Published:** 2023-05-07

**Authors:** M. Bordiga, C. Lupi, G. Langer, A. Gianoncelli, G. Birarda, S. Pollastri, V. Bonanni, D. E. Bedolla, L. Vaccari, G. Gariani, F. Cerino, M. Cabrini, A. Beran, M. Zuccotti, G. Fiorentino, M. Zanoni, S. Garagna, M. Cobianchi, A. Di Giulio

**Affiliations:** 1 National Institute of Oceanography and Applied Geophysics-OGS, Via Auguste Piccard 54, 34151 Trieste, Italy; 2 Department of Earth and Environmental Sciences, University of Pavia, Via Ferrata 1, 27100 Pavia, Italy; 3 ICTA, Autonomous University of Barcelona (UAB), 08193 Bellaterra, Spain; 4 Elettra-Sincrotrone Trieste, Strada Statale 14, km 163.5 in Area Science Park, 34049 Trieste-Basovizza, Italy; 5 AREA Science Park, Padriciano 99, 34149 Trieste, Italy; 6 Department of Biology and Biotechnologies “Lazzaro Spallanzani”, University of Pavia, Via Ferrata 9, 27100 Pavia, Italy

**Keywords:** Biogeochemistry, Marine biology, Palaeontology

## Abstract

Coccolithophores, marine calcifying phytoplankton, are important primary producers impacting the global carbon cycle at different timescales. Their biomineral structures, the calcite containing coccoliths, are among the most elaborate hard parts of any organism. Understanding the morphogenesis of coccoliths is not only relevant in the context of coccolithophore eco-physiology but will also inform biomineralization and crystal design research more generally. The recent discovery of a silicon (Si) requirement for crystal shaping in some coccolithophores has opened up a new avenue of biomineralization research. In order to develop a mechanistic understanding of the role of Si, the presence and localization of this chemical element in coccoliths needs to be known. Here, we document for the first time the uneven Si distribution in *Helicosphaera carteri* coccoliths through three synchrotron-based techniques employing X-ray Fluorescence and Infrared Spectromicroscopy. The enrichment of Si in specific areas of the coccoliths point to a targeted role of this element in the coccolith formation. Our findings mark a key step in biomineralization research because it opens the door for a detailed mechanistic understanding of the role Si plays in shaping coccolith crystals.

## Introduction

**Figure 1:**
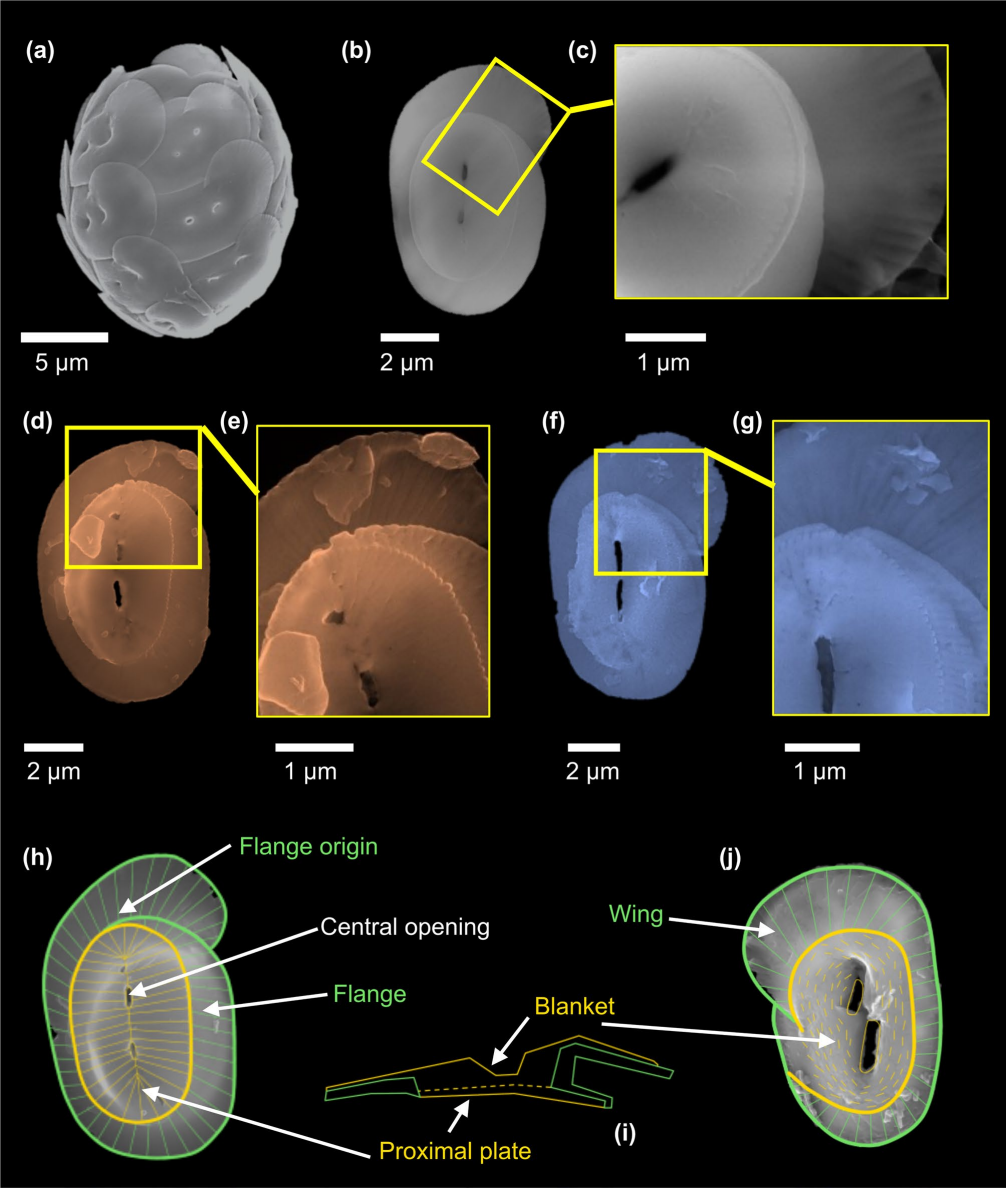
Micrographs of the species *Helicosphaera carteri* at scanning electron microscope (SEM). Micrographs collected from monospecific culture C1: (**a**) entire coccosphere (10,000× magnification), (**b**) single coccolith (25,000×), and (**c**) detail on calcite element structure of a coccolith (60,000×). Images of fossil remains of *H. carteri* picked from sample F1 (**d**–**e**) and F2 (**f**–**g**): single coccoliths (**d** 30,000×; **f** 20,000× magnification); particulars of calcite crystal arrangement (**e** 55,000×; **g** 60,000×). Scale bars are reported for all micrographs. Scheme of *H. carteri* coccolith structure and specific terminology redrawn and modified from Young et al.^22^: (**h**) proximal, (**i**) section, and (**j**) distal views. Coccolith micrographs behind the schemes are from sample F2: (**h**) *H. carteri*, (**j**) *H. wallichii*.

Coccolithophores are marine unicellular organisms covering their cell with calcium carbonate (CaCO_3_) platelets—the coccoliths—forming an exoskeleton called coccosphere (Fig. 1a,b)^1,2^. Since coccolithophores play a pivotal role in the global carbon cycle through both the organic carbon pump and the carbonate counter pump^3,4^, understanding their physiology is fundamental to predict their future distribution and abundance in the oceans, and thus their role in carbon fixation and export. Until recently, the study of coccolithophore calcification physiology has mainly focused on the structural components of coccolith calcite, i.e. calcium ion (Ca^2+^) and dissolved inorganic carbon^1,5,6^, and on the fractionation of certain minor elements such as strontium (Sr)^7–9^. Since silicon is not a component of calcite, the Si requirement of some coccolithophore species had not been expected and was not realized until Durak et al.^10^ showed that some species need Si to calcify. These Si-dependent species express diatom-like silicate transporters (SITL) emphasizing the nutrient-role played by Si^10^. Considering the counter-intuitive notion of a Si-dependence in the calcite producing process (coccolithogenesis) it is by no means self-evident why some coccolithophores should need Si to survive. A clue might be gleaned from the example of *Coccolithus braarudii* that needs a coccosphere for cell division^11^. An intact coccosphere depends on normally formed coccoliths^12^, and normal coccolith morphogenesis in turn depends on Si in this species^11^. More specifically, it was shown that heterococcolith (HET) crystal morphogenesis is a Si-dependent process, in particular crystal growth modification, as opposed to nucleation^13^. Understanding crystallization and crystal shaping are at the heart of biomineralization and biomimetics (crystal design) research. Despite this centrality of coccolith crystallization and shaping, these processes are poorly understood^14^. The requirement of Si in some coccolithophore species opens up a novel way of looking at the mechanism of biomineral morphogenesis. Research is in its infancy and fundamental data such as the presence and distribution of Si in coccoliths are still missing.

In this study, we document for the first time the presence and localization of Si within both fossil and cultured coccoliths of the *Helicosphaera carteri* species (Fig. 1). This species was selected as it may be Si-dependent since it belongs to the order of the Zygodiscales which includes *Scyphosphaera apsteinii*, a species using SITLs^10,13^. The physiology of *Helicosphaera carteri* has been little studied so far, although it is prolific in both open ocean and estuarine/neritic areas^15–17^ and it has an abundant geological record since emerging in the Early Miocene (ca. 23 Ma)^18^. It also strongly contributes to the marine CaCO_3_ production and carbon storage both in past^15,19^ and present^4,20,21^ sediments. *Helicosphaera* has a unique morphology characterized by: (i) a spiral flange developing along the elliptical margin of the coccolith, (ii) a blanket forming the distal cover, (iii) a proximal plate composed of radial discrete elements, and (iv) two central openings^22^ (Fig. 1h–j).

To detect the presence of Si trace amounts within coccoliths, and also its content and distribution, we applied three synchrotron-based high-resolution and non-destructive techniques at Elettra Sincrotrone Trieste synchrotron radiation facility: (i) X-ray Fluorescence (XRF) at the XRF beamline; (ii) Scanning Transmission X-ray Microscopy (STXM) coupled with Low Energy XRF (LEXRF) at the TwinMic beamline; (iii) Fourier Transform Infrared Spectromicroscopy (μFTIR) at the Synchrotron Infrared Source for Spectroscopy and Imaging (SISSI-Bio) beamline. Although the use of these advanced and complementary analytical techniques is becoming more widely employed in the environmental and life science fields, as well as in investigating the biomineralization of living and fossil organisms^23–25^, they have been little exploited for coccolithophore studies. So far, only a few works investigating trace elements contained in cultured and fossil coccoliths employed synchrotron-based X-ray fluorescence^26–29^ or molecular spectroscopies^30–32^. These studies were mainly focused on chemical elements heavier than Si, namely calcium (Ca), iron (Fe), zinc (Zn), and Sr, thus the Si presence was not studied and was only briefly mentioned in one study^29^. By using specific instrument settings and suitable energy ranges at the three synchrotron beamlines, we document for the first time not only the presence but also the localization of Si in both fossil and cultured coccoliths of *H. carteri*.

## Results

### Silicon localization in coccoliths identified through synchrotron-based XRF analyses

**Figure 2:**
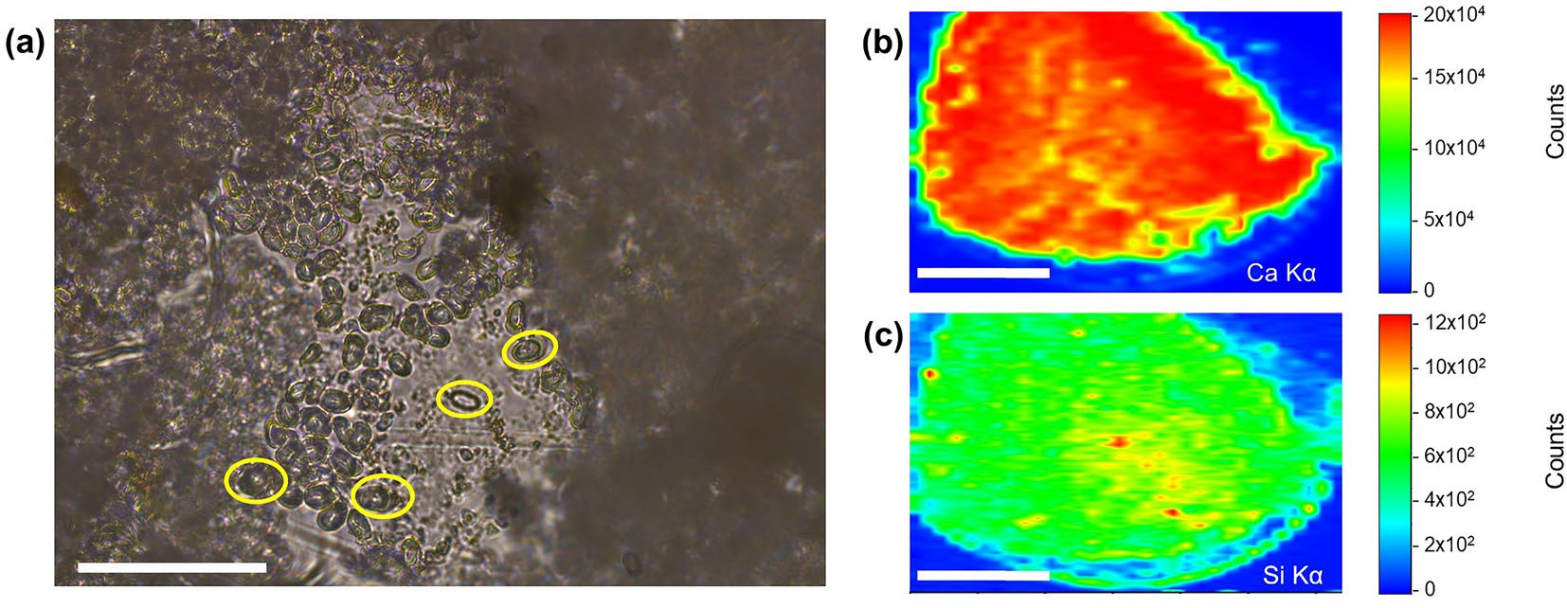
Data collected at XRF beamline on coccoliths picked from the monospecific culture of *H. carteri* (C1). (**a**) Micrograph acquired at light microscope in transmission mode at 400× magnification. Some coccoliths are highlighted by yellow circles in the less dense area of the deposited sample. Scale bar is 50 μm. XRF maps of (**b**) Ca Kα and (**c**) Si Kα lines (slightly smoothed) collected with a 5 keV incident beam, highlighting their co-localization; spatial resolution is equal to 50 × 200 µm^2^. Scale bars are 2 mm. Color scale bars are also reported, with the counts normalized to the incident I0 intensity. Maps were generated with PyMCA software package^36^ (https://pymca.sourceforge.net/).

**Figure 3:**
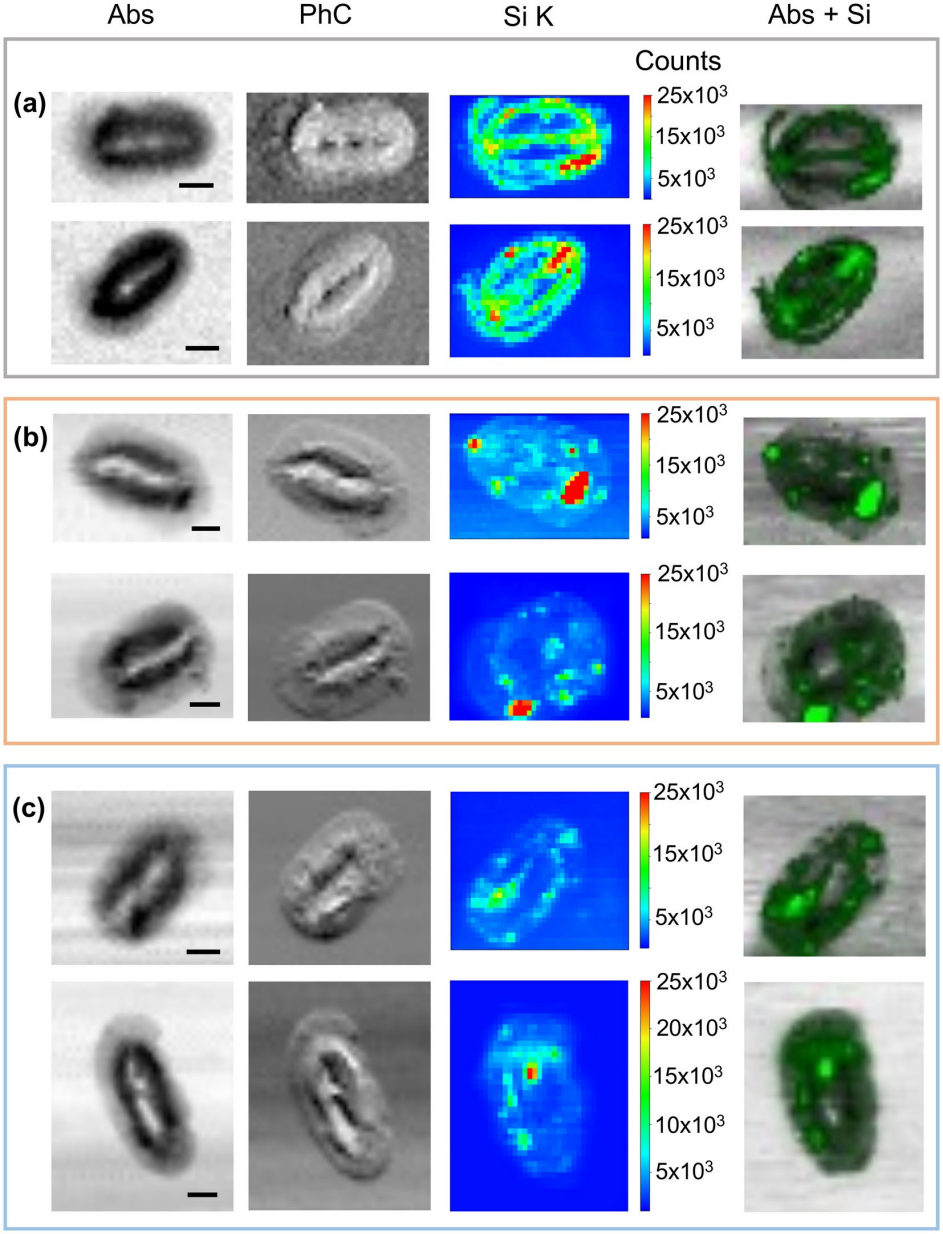
Examples of XRF maps collected at TwinMic on single coccoliths of *H. carteri* from monospecific culture sample C1 (**a**), and fossil samples F1 (**b**) and F2 (**c**). Absorption (Abs) and phase contrast (PhC) images of coccoliths are depicted together with the corresponding Si XRF map (Si_K) and the overlapping image of absorption and Si distribution (Abs + Si). All images were acquired at 1.92 keV with 300 nm step size and 60 ms acquisition time for Abs and PhC, while 15 s for XRF map. Scale bar is 2 μm. Color bars report the intensity counts. Maps were produced using PyMCA software package^36^ (https://pymca.sourceforge.net/).

The XRF maps, acquired on a concentrated amount of cultured coccoliths of *H. carteri* (sample C1; Fig. 2a), evidenced at micrometric resolution not only a high signal of Ca, which is well-known to be one of the main components of coccoliths^33–35^ (Fig. 2b), but also the presence of Si (Fig. 2c). For monospecific fossil samples (F1 and F2), the measurements at single coccolith level were difficult due to the beam size (> 20 µm) of XRF beamline, but yet XRF maps revealed the overlap of Si and Ca (Fig. S1). Once identified the presence of Si at the XRF beamline, the Si signal was more thoroughly investigated at a sub-micrometric length scale, thus at single coccolith level, using TwinMic beamline. LEXRF mapping on 13 single coccoliths of *H. carteri* indubitably confirmed not only Si presence in all samples, both cultured (C1) and fossil (F1 and F2), but also revealed its localization on single coccolith (Figs. 3, S2). All the maps are well comparable among the different types of samples, returning similar absorption (Abs) and differential phase contrast (PhC) images with lower transmitted signals in the internal portion of the coccolith, which is also the thicker part of this species morphology, where the proximal plate overlaps with the blanket (Figs. 3, S2). The overlay of LEXRF maps of Si (Si_K) with the corresponding absorption images (Abs + Si) allowed to highlight Si localization and to compare it with the coccolith morphology. Overall, in cultivated coccoliths Si seems to be located along the flange and the margin of the proximal plate, as well as along the long axis crossing the central opening(s) (Figs. 3a, S2a). Some Si concentrated accumulations on a specific area of the external margins are also noted, but they are not necessarily associated with a visible feature on the absorption or phase contrast images. Fossil coccoliths show a less uniform or characteristic Si distribution compared to the cultured ones. Silicon appears to be located more as hot spots, sometimes visible as darker areas in the absorption images, thus possibly related to the presence of small pieces belonging to other fossil coccoliths (Figs. 3b,c, S2b,c). In such areas, Si is reaching much higher levels than in the overall cultivated coccoliths, while the area of the proximal plate of the fossil *H. carteri* appears on average more depleted in Si than the corresponding area of C1 coccoliths.

### Chemical bonds detected by Infrared Spectromicroscopy analysis

**Figure 4:**
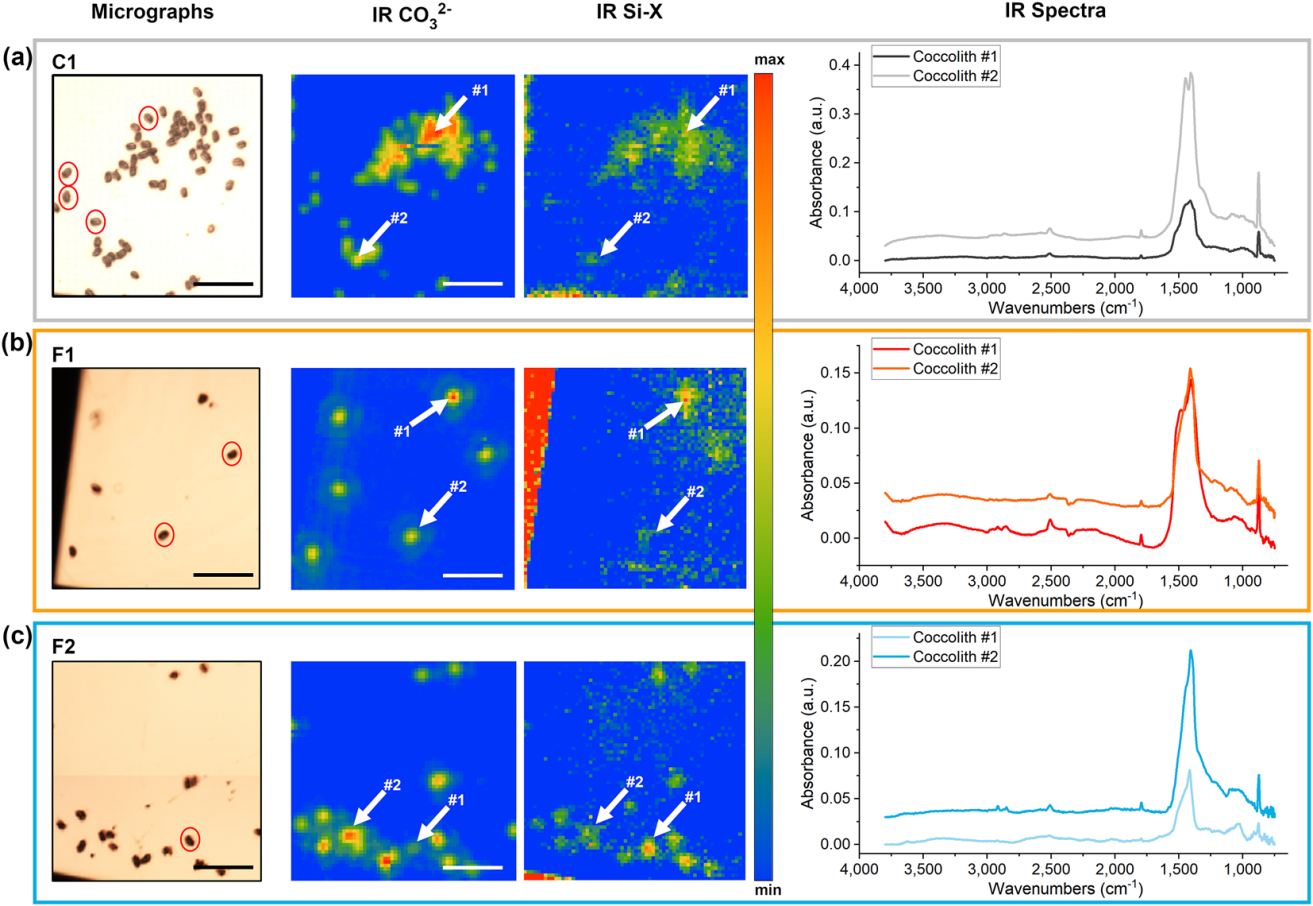
Examples of micrographs, CO_3_^2−^, and Si-X maps collected through SISSI-Bio beamline on single coccoliths of *H. carteri* isolated from monospecific culture sample C1 (**a**), and fossil samples F1 (**b**) and F2 (**c**). Some of the coccoliths analyzed at both SISSI-Bio and TwinMic beamlines are highlighted with red circles. Scale bar is 50 μm. Reported spectra are derived from the coccoliths named #1 and #2 and highlighted by the white arrows. In the spectra, the crystalline form of CaCO_3_ is ascribable to the 1600–1300 cm^−1^ broad band and the peak at 865 cm^−1^; whereas the Si chemical bonds with other elements are identifiable by the band from 1200 to 950 cm^−1^ with a peak at 1075 cm^−1^. Maps and spectra were generated using Quasar software^37^ (https://quasar.codes/).

While X-ray Fluorescence technique clearly reveals Si presence, it does not provide information on the chemical environment surrounding it. Infrared Spectromicroscopy at the SISSI-Bio beamline, on the other hand, allowed us to identify peaks related to the vibrations of Si-X, where X may be oxygen, carbon or nitrogen. The returned maps of CO_3_^2−^ ascribable to the presence of the crystalline form of CaCO_3_ show a good correspondence with Si-X distribution demonstrating that both μFTIR signals co-localize with the coccoliths (Fig. 4). A total of 246 μFTIR spectra acquired at micrometric resolution on single coccoliths complement the results on Si signals obtained at TwinMic at nanometric resolution on a smaller number of coccoliths (Table 1). The variable intensity of CO_3_^2−^ and Si-X maps and the μFTIR spectra from individual coccoliths document a high variability within the same sample (See IR-spectra in Fig. 4).

## Discussion

### Measurement of Si in coccoliths

This study shows for the first time the peculiar localization of Si in both cultured and fossil coccoliths of *H. carteri*. We can exclude any contamination of the Si signal derived from coccoliths since: (i) sample supports and sample holders were intentionally selected to be Si-free (i.e. Mylar films and Au TEM grids with Formvar); (ii) Si concentration of the seawater used for the culture medium was low (0.85 μM), even though not limiting for coccolith growth^13^ and, in addition, the culture sample was thoroughly cleaned to completely remove the medium before the analyses; (iii) Si was recorded in both cultured and fossil samples, and therefore is unlikely to derive from any specific of the medium (for the culture) nor any depositional/post-depositional process (for the fossils). For the fossil samples, we can rule out that the Si signal may be derived from the presence of colloidal silica since they are not recorded in the North Pacific at any depth of the water column or at the water–sediment interface^38^.

We were able to clearly detect Si and describe its localization because of the different instrumentation and settings compared to previous synchrotron-based studies on coccolithophores. In fact, Bottini et al.^26^ and Suchéras-Marx et al.^28^ performed XRF measurements in air in the hard X-ray regime by using an excitation energy of 17 keV, far from the Si absorption K-edge, thus impairing Si detection (Table 1). Silicon XRF photon detection requires vacuum or a thin layer of air between sample and detector. Only Suchéras-Marx et al.^29^ used an in-vacuum setup with a lower incident energy (7.5 keV) identifying some Si peaks in the spectra of fossil coccoliths of *Coccolithus pelagicus*, *H. carteri* and *Calcidiscus leptoporus*, although no attention was paid to Si as its signal was very weak (Table 1). Here, both XRF and TwinMic beamlines provided optimal excitation for Si by applying lower energies, closer to Si absorption K-edge and also aided by vacuum conditions (Table 1). Finally, the acquisition via μFTIR of a distinctive Si-X signal calculated from the spectra of 246 single coccoliths isolated from both cultured and fossil samples, statistically corroborates the Si presence within the mineralized fraction of *H. carteri.* The high number of individual coccoliths analyzed at TwinMic and SISSI-Bio beamlines together with the statistical approach carried out at SISSI-Bio allowed to highlight the intraspecific variability. This could not be evaluated in other pilot studies, where a single or a very small number of coccoliths were analyzed^26,28,29^ (Table 1). By combining the data, we could unambiguously identify the presence of Si within both cultivated and fossil coccoliths of *H. carteri* together with its distribution in the coccoliths from micrometric to nanometric resolution on a high number of coccoliths never analyzed before, especially for the fossil record (Table 1).

**Table 1:**
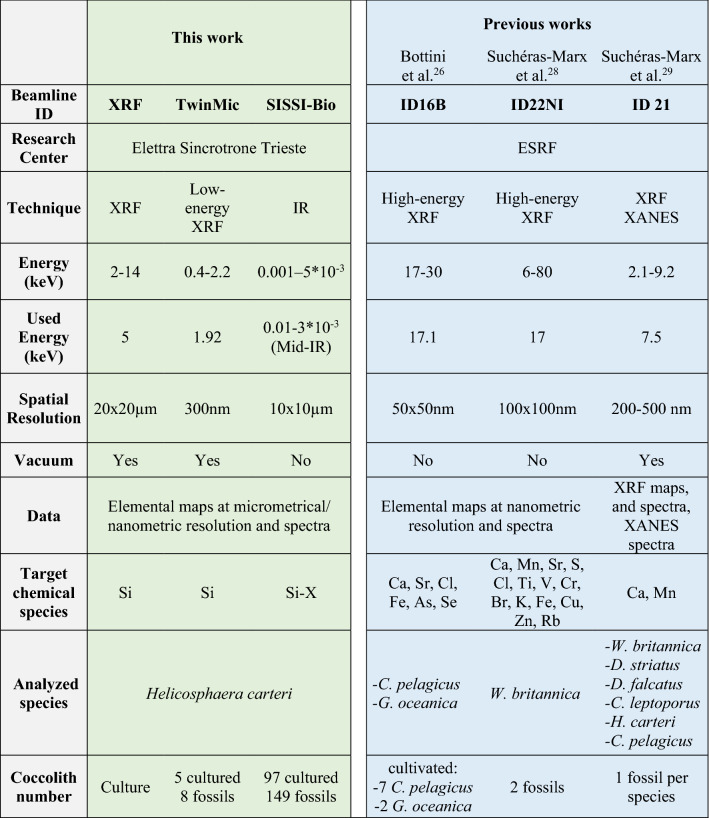
Specifics of the Elettra Sincrotrone Trieste beamlines employed in the present work, compared with those of the European Synchrotron Radiation Facility (ESRF) beamlines used in previous coccolith-related works. XRF, X-ray Fluorescence; IR, Infrared Spectromicroscopy and Imaging.

### Localization and potential function of Si in coccoliths

The surprising discovery of a Si requirement in some coccolithophore species has immediately posed the question as to the function of Si in coccolithophore biology. The seminal study by Durak et al.^10^ proposed a role of Si in calcification. Although counterintuitive, a subsequent study confirmed this inference but left the question of the specific role of Si in the multi-facetted calcification process open^11^. More recently, it was suggested that Si plays a specific role in the shaping of HET coccolith crystals^13^. Therefore, normal HET coccolith morphogenesis depends on Si in Si-requiring species such as *S. apsteinii*. Coccolith morphogenesis in general and crystallization/crystal shape control in particular remain enigmatic processes^39^. In order to better understand the mechanism of coccolithogenesis, it is important to obtain information about the presence and distribution of minor elements as well as organic compounds in coccoliths^14^. While the distribution of minor elements in general is helpful, the one of Si is particularly important because Si plays a role in morphogenesis on Si-requiring species, contrary to other minor elements such as Sr^7^. Unfortunately, very little is known about the presence of Si in coccoliths, and even less about its distribution. The data presented here clearly show that Si is present in coccoliths of *H. carteri*. Although we did not quantify Si content, we found Si to be below the detection limit of the Energy-dispersive X-ray spectroscopy (EDS) (Fig. S3). This observation tallies well with other EDS^40,41^ and ICP-MS measurements showing that Si/Ca in *C. leptoporus*, *C. braarudii*, and *E. huxleyi* is in the µmol/mol range^41^. The low Si content of coccoliths provides important clues as to the function of Si in morphogenesis. Rather than being a structural component of coccolith calcite, Si seems to play a modificatory role^13^. It is unknown which component of the morphogenetic machinery uses Si. These components are nucleation control through an organic template, and crystal growth control^42^. The latter is achieved through inorganic crystal growth, cytoskeletal pull, and interaction of the growing crystals with coccolith-associated polysaccharides (termed CAPs) or other organic molecules. Based on a detailed analysis of Ge-induced coccolith malformations the target process of Si action was narrowed down to crystal growth control^13^. There is also morphological evidence against a Si-cytoskeleton interaction, since Ge-specific malformations are not observed in response to cytoskeleton-inhibitor treatment^13,43^. Therefore, a role for Si in intra-coccolith vesicle (intra-CV) CAP or protein functioning is most plausible. In *S. apsteinii*, a close relative of *H. carteri*, the Si requirement seems to be confined to certain stages of lopadolith growth^13^.

If Si plays a specific role in certain stages of coccolith growth an uneven distribution within the coccolith might be expected. We do indeed observe such an uneven distribution of Si in *H. carteri*. On average, higher concentration of Si is detectable along the margin of the flange and the proximal plate together with the central axial line (Fig. 3a).

At this point it is helpful to compare coccolithophore biomineralization with that of extracellular calcifiers such as foraminifera and molluscs. The latter feature a well described layered growth resulting in an uneven distribution of organic material and minor elements^25^. The uneven distribution of minor elements has a characteristic banding pattern relating to the growth layers. This banding pattern does not point to a specific role of a minor element in morphogenesis but is mostly the accidental by-product of different fractionation steps involved in the formation of growth layers. Coccolithophore biomineralization, by contrast, is thought to proceed via a fundamentally different mechanism. After controlled nucleation on the base plate, crystal growth is thought to proceed in an inorganic fashion modified by shape control through the cytoskeleton and intra-CV organic material, chiefly CAP^42^. This growth mechanism is not expected to produce a minor element banding pattern^14^. Current process-based fractionation models predict an even distribution of minor elements instead^44–46^. Modification of these models to accommodate an uneven distribution is possible but requires further assumptions^7^.

The distribution of Si in *H. carteri* reported here clearly deviates from both an even distribution and a banding pattern. From the observed Si enrichment in certain parts of the coccolith two important conclusions can be drawn. Firstly, Si does not merely follow the transport pathway of other ions, but has its own transport system enabling the specific local Si enrichment. Secondly, process-based fractionation models describing Si incorporation will need to include this Si transport system and find a way of accounting for the observed Si distribution. This targeted Si transport supports the notion that Si plays a role in certain stages of coccolith growth in *H. carteri*.

Since our approach resolves the Si signal only in the x–y-plane (shield plane) of the coccolith and integrates the signal along the z-axis (perpendicular to the shield plane) we cannot locate Si on the z-axis. Consequently, we do not know whether Si is evenly distributed along the z-axis. By entailment this means that Si could reside in the crystal lattice or in organic material, or both. These options are also supported by Si bonding to C, O, or N, as inferred from our infrared data. Please note that our interpretation that Si plays a role in certain stages of coccolith formation does rest on its uneven distribution in the shield-plane alone. To our inference the precise nano-localization of Si is irrelevant. It would, however, be helpful to know whether Si resides in the calcite lattice or in organic material, because this knowledge would provide further clues as to the precise mechanism of action of Si in morphogenesis. Here, we provide evidence supporting the idea that Si plays a role in morphogenesis, and that this role might be confined to certain stages of coccolith growth.

### Si in fossil coccoliths

The discovery of Si in fossil coccoliths demonstrates that this element is not easily removed over time and can be identified even in the geological record. In our fossil samples, the spatial distribution is less regular, showing the presence of preferential points of concentration (Fig. 3b,c). The latter could be due to the influence of fossilization and sedimentation processes slightly affecting the coccoliths’ preservation. More specifically, as the fossil samples F1 and F2 derive from young (118,000 and 138,000 years ago respectively^19^) and semi-consolidated sediments drilled in the first 1.5 m below sea floor^47^, the processes acting on coccoliths can be mainly related to: (i) biostratinomy that involves the coccoliths’ disarticulation from the cell, their dispersal in the water column, the accumulation into the sediments, and the fossilization; (ii) and pre-diagenesis mainly detectable as chemical carbonate dissolution for the studied samples. Between the two fossil samples, F2 returns LEXRF maps more comparable to C1 than F1 (Figs. 3b,c; S2b,c). This evidence could be related to the carbonate dissolution which has been previously documented to be stronger in F1 than F2^48^ (Fig. 1b–g), although the overall preservation of the fossil assemblages is on average very good^19,47,48^.

Despite the spotted concentration of Si in the fossil samples, its distribution is nevertheless similar to the one of the cultured coccoliths since the hot spots are localized mainly along the flange, the margins, and the central axis (Fig. 3b,c). Our hypothesis that Si may be bound to organic components remains plausible since CAPs can be found in fossil coccoliths up to 70 Ma^49^, and the presence of amino acids has been documented on fossilized coccoliths of *C. leptoporus* from 470,000 years ago^50^. In the present study, our oldest sample is dated 138,000 years, thus not old enough, but Si XRF maps of fossilized coccoliths older than 70 Ma are planned and will prove helpful here to verify the Si-CAPs bond hypothesis. If the latter is correct, it is possible that in *H. carteri* Si, thus CAPs, could be more concentrated on the flange where many crystals compose the complex external portion, whereas the central area constituted by larger and less numerous crystals^22^ retains less silicon. Regarding the Si-proteins bond, to date there is no evidence on the preservation of protein or amino acid in coccoliths beyond 470,000 years ago^50^, unlike for other biomineralized fossil organisms^51^.

## Conclusion

Our data show through an innovative multi-technique approach the potential of synchrotron X-ray Fluorescence and Infrared Spectromicroscopy to identify the presence and localization of Si in both cultured and fossilized coccoliths. The high number of individual coccoliths analyzed renders our results statistically robust in a way never achieved before in coccolithophore studies. We document for the first time that Si is unevenly distributed in the coccoliths of the species *H. carteri*, with the flange margin, proximal plate, and central axial line enriched in Si. This distribution is best explained by a specific role for Si in certain stages of coccolith growth. Our data do not allow a precise nano-localization of Si along the z-axis (perpendicular to the shield plane), neither can we infer whether Si resides in the calcite lattice or the organic material that is part of the coccolith. Future studies should aim to answer these questions since they have a bearing on the precise molecular mechanism of Si action.

This knowledge is also central to developing Si fractionation models. Such a mechanistic understanding, in turn, is likely to significantly advance our knowledge of biomineralization in general and crystal design for biomimetic and technological applications. Mechanistic understanding is also necessary to assess the ecophysiology of marine calcifiers and this will eventually enable us to make better predictions about their fate under climate change.

## Methods

### Fossil sample preparation

The fossil remains of *Helicosphaera carteri* analyzed here come from marine deep-sea sediments collected at the Ocean Drilling Program (ODP) Site 1209B drilled at a water depth of 2387 m on the Shatsky Rise, a marine plateau located at 1600 km eastward from Japan (NW Pacific Ocean). The sedimentary sequence consists of nannofossil oozes, i.e. fossil remains of coccolithophores, with some other minor components such as foraminifera, diatoms, radiolaria and clay^47^. From this sequence, two samples were selected: sample F1 was drilled at 1.1 m below sea floor (mbsf), deposited during the last interglacial interval called Marine Isotope Stage (MIS) 5 and dated 118,000 years ago^19^; sample F2 was collected at 1.4 mbsf from the foregoing glacial phase MIS 6 (dated 138,000 years ago)^19^. At the Department of Biology (University of Pavia), single coccoliths of *H. carteri* were individually picked from the sediment with a micromanipulator Olympus IX71 following Bordiga et al.^52^. The big-sized coccoliths of *H. carteri* made the picking process more manageable. For synchrotron-based measurements, at least 100 coccoliths per sample were placed on an Au TEM grid with Formvar layer in order to be analyzed at TwinMic and SISSI-Bio beamlines, while additional 100 coccoliths were deposited between two layers of Mylar film for analysis at XRF beamline. The coccolith preservation and structure were investigated through the acquisition of micrographs at the Scanning Electron Microscope (SEM) Tescan Mira 3XMU at the “Arvedi” CISRic Laboratory of the Department of Earth and Environmental Sciences (University of Pavia).

### Culture setup and sample preparation

Monoclonal cultures of the species *Helicosphaera carteri* (strain RCC1323 from the Roscoff Culture Collection) were grown in the B medium (cosmi.ogs.it/node/7) obtained from the natural seawater collected in the Gulf of Trieste (northern Adriatic Sea, Italy) after filtration through 0.22 μm pore size Durapore membrane filters (Millipore), autoclaving, and supplement of nutrients (nitrate and phosphate), metals and vitamins. The natural content of silicon in the collected seawater was 0.825 μmol L^−1^ [M. Kralj, personal communication], close to the value < 1.6 μmol L^−1^ measured on a station close to the ODP Site 1209^53^. The experiments were conducted in 2.5L-photobioreactors (Kbiotech) controlled by the BioFlex software at the National Institute of Oceanography and Applied Geophysics (OGS) in Trieste. The environmental conditions documented for MIS 5^54,55^ were reproduced during the experiment by keeping stable the following parameters: CO_2_ 290 ppm, temperature 19 °C, salinity 35 PSU, light irradiance 100 µmol m^−2^ s^−1^, and light/dark cycle of 12:12 h mimicking the latitudinal day/night regime for the subtropical Site 1209 studied here in the fossil record. A pitched-blade impeller guaranteed culture agitation. The strain was acclimated to the experimental conditions for two weeks before inoculation. For statistical relevance, three independently grown replicates were carried out. As suggested by La Roche et al.^56^ coccospheres were sampled during the exponential phase but still with low cell density (≤ 10,000 cells mL^−1^) to prevent a significant change in the dissolved inorganic carbon (DIC) in the medium and a strong shift of the medium pH. Coccoliths were separated from the cells by adding a solution of buffered MilliQ and Triton at 1%. For the final sample cleaning three rinses were carried out with MilliQ buffered with ammonia (10%, pH ≥ 10) to prevent dissolution. For analyses at the synchrotron facilities, an aliquot of coccolith suspension was placed on an Au TEM grid with Formvar layer, whilst another denser aliquot was deposited between two Mylar films. All samples derived from the culture experiment are labeled as C1. The coccosphere and coccolith micrographs were collected via SEM Tescan Mira 3XMU at “Arvedi” CISRic Laboratory of the Department of Earth and Environmental Sciences (University of Pavia, Italy). The SEM was also equipped with the EDAX EDS detector for carrying out analyses via Energy Dispersive Spectroscopy (EDS) on a coccolith from C1 to detect the silicon. The EDS measurements were processed with EDAX Spectrum Viewer software (https://www.edax.com/support/spectrum-viewer).

### X-ray fluorescence (XRF) beamline

X-ray Fluorescence is a multi-element technique which returns the spatial distribution of chemical elements. In this study, both cultured and fossil coccoliths were investigated at the XRF beamline of Elettra Sincrotrone Trieste (Italy)^57^. Specifically, the XRF beamline covers the energy range from 2 to 14 keV, allowing detection of element fluorescence K lines from Na to Br (and L lines up to Po) with a lateral spatial resolution > 20 µm (Table 1). For analyses at XRF beamline both the fossil and culture samples were deposited between two layers of Mylar film, sealed with a Delrin interlocking ring and fixed on a dedicated Al sample holder. The Mylar films are composed of polyethylene terephthalate, thus do not contain silicon. The measurements were conducted using the Medium Energy (ME) multilayer monochromator for the collection of maps at micrometric resolution with an incident beam energy of 5 keV (Table 1). A varying beam size at the exit slits was selected depending on the sample area ranging from 200 × 200 µm^2^ to 20 × 20 µm^2^, and with a standard 45°/45° geometry for fluorescence mode measurements, using an XFlash 5030 Silicon Drift Detector (SDD) detector (Bruker, Berlin, Germany). Higher order harmonics contamination was suppressed by a pair of parallel plane mirrors intercepting the beam in grazing incidence. All XRF spectra were processed with the PYMCA software package^36^ (https://pymca.sourceforge.net/).

### Soft X-ray microscopy and low energy X-ray fluorescence—TwinMic

MicroXRF analyses were performed at the TwinMic beamline at Elettra Sincrotrone Trieste (Italy)^58^, which works in the 0.4–2.2 keV energy range (Table 1). Both fossil and culture samples were deposited on Au TEM grids covered with a Formvar film, which has an exceptional radiation tolerance and is silicon-free. A total of 13 low energy XRF (LEXRF) maps of *H. carteri* single coccoliths were collected (5 for C1, 4 for F1, and 4 for F2) for around 100 h of analyses (ca. 8 h per coccolith). The selected 13 coccoliths are a subset of the coccoliths analyzed by infrared spectromicroscopy at SISSI-Bio beamline, allowing a direct comparison on the same coccoliths. To avoid any possible radiation damage due to soft X-rays, the coccoliths were firstly measured at SISSI-Bio beamline as Infrared (IR) spectromicroscopy is not damaging and label free. By applying these two analytical techniques we can provide complementary information allowing us to get a more complete overview of the specimen’s chemistry. In the present experiments, the TwinMic microscope was operated in Scanning Transmission mode with an incident beam energy of 1.92 keV to get optimal excitation of silicon. A 600 µm diameter Au zone plate diffractive optics with a 50 nm thick outermost zone produced a 350 nm diameter beam perpendicularly incident on the sample plane. The coccoliths were raster scanned across the microprobe with a step size of 300 nm. For each pixel in the raster scan a fast readout CCD camera collected the transmitted X-ray photons generating absorption and phase contrast images, delineating coccolith morphology; simultaneously 8 SDDs, located at 20° from the sample plane and 28 mm from the sample, acquired the XRF photons emitted by the sample, producing XRF elemental maps with nanometric spatial resolution. All XRF spectra were processed and batch fitted with the PYMCA software package^36^ (https://pymca.sourceforge.net/).

### Synchrotron infrared source for spectroscopy and imaging—SISSI-Bio

The same samples C1, F1, and F2 deposited onto Au TEM grids were measured at the Synchrotron Infrared Source for Spectroscopy and Imaging (SISSI-Bio) beamline at Elettra Sincrotrone Trieste (Italy)^59^, before being exposed to soft X-rays at TwinMic beamline. If X-ray beamlines both provide a chemical characterization at elemental level, the analysis at SISSI-Bio returns Fourier-transform infrared spectromicroscopy (µFTIR) data that can give information at molecular level, identifying the chemical moieties present within the sample. Photons in the Mid-infrared range (4000–400 cm^−1^, 0.496–0.0496 eV) have energies that corresponds to those of the common covalent bonds present in organic samples, like C–O, N–O, C–H, N–H, P–O, C–N, but also to those of some inorganic covalent bonds like Al–O, Si–O, Fe–O, Si–N, Si–H. Commonly, FTIR spectroscopy is a bulk technique that provides average chemical information on large areas, but thanks to the brightness of Synchrotron Radiation, it is possible to focus the infrared (IR) beam at the diffraction limit, i.e. a few micrometers for the medium infrared (MidIR) regime, and to collect spectra from single coccoliths. Moreover, being a broadband technique, the whole range can be registered at the same time, for each point. Switching from spectromicroscopy to Fourier Transform IR imaging, it was then possible to acquire hyperspectral infrared images of the distribution of specific chemical species within the inspected sample. By combining the data acquired with the two modalities it is possible to: (i) identify the bands of interest with the spectroscopic approach from each single coccoliths, and (ii) obtain images of chemical distribution within the coccoliths by integrating those signals. Spectra from individual coccoliths were collected using the VIS-IR microscope Hyperion 3000 (Bruker Optics, Billerica, Ma, US) coupled with a VERTEX 70 V in-vacuum interferometer (Bruker Optics, Billerica, Ma, US), using Synchrotron IR emission (SRIR) as a source and a 100 µm MCT (Mercury-Cadmium-Telluride) detector and averaging 512 scans at 4 cm^−1^ spectral resolution in transmission mode, i.e. the light will pass through all the thickness of the sample and then read by the detector. A 15X cassegrain objective-condenser pair was used and the view-through apertures were set at 10 × 10 µm in order to collect the signal from individuals. Samples were also imaged using a 64 × 64 bidimensional array detector (FPA, Focal Plane Array) through the same optics (15X, in transmission mode as well) therefore with a pixel size of 2.62 µm over an area of 167 × 167 µm. Data were corrected for water vapor using OPUS 8.5 SP1 (Bruker Optics, Billerica, Ma, US) and then analyzed using QUASAR software^37^ (https://quasar.codes)^57^. Band integrals were calculated in the range of calcium carbonate (1800–1700 cm^−1^ and 1600–1300 cm^−1^), and silicate/phosphates (1200–1000 cm^−1^). The same spectral ranges were used to generate spectral maps. The chemical distribution maps of CO_3_^2−^ have been generated by integrating the 1600–1300 cm^−1^ broad band and the peak at 865 cm^−1^ which are ascribable to the presence of the crystalline form of CaCO_3_^60^. The maps representative of the Si chemical bonds with other elements are derived from the integration of a second band from 1200 to 950 cm^−1^ with a peak at 1075 cm^−1^, that can have manifold attributions: from C–O–C vibrations from carbohydrates^30^, to vibration of phosphates^32^, to silicate vibrations^60^ and some even to strontium carbonate^61^. This attribution difficulty may be overcome by the support of the other two XRF techniques. A total of 246 coccoliths were analyzed: 97 for the sample C1, 50 for F1, and 99 for F2. This amount of infrared spectral data from single coccoliths has never been achieved before, especially in fossil samples (Table 1).

## Supplementary Information

Supplementary Information.

## Data Availability

All data needed to evaluate the conclusions in this work are present in the paper and/or in the Supplementary information.
