# Supplementary material for: Unexpected silicon localization in calcium carbonate exoskeleton of cultured and fossil coccolithophores
Source: Sci Rep. 2023 May 7;13:7417. doi: 10.1038/s41598-023-34003-3 (PMC10164752; doi:10.1038/s41598-023-34003-3)
Supplement: Supplementary file 1 — Supplementary Information. [file 41598_2023_34003_MOESM1_ESM.pdf]

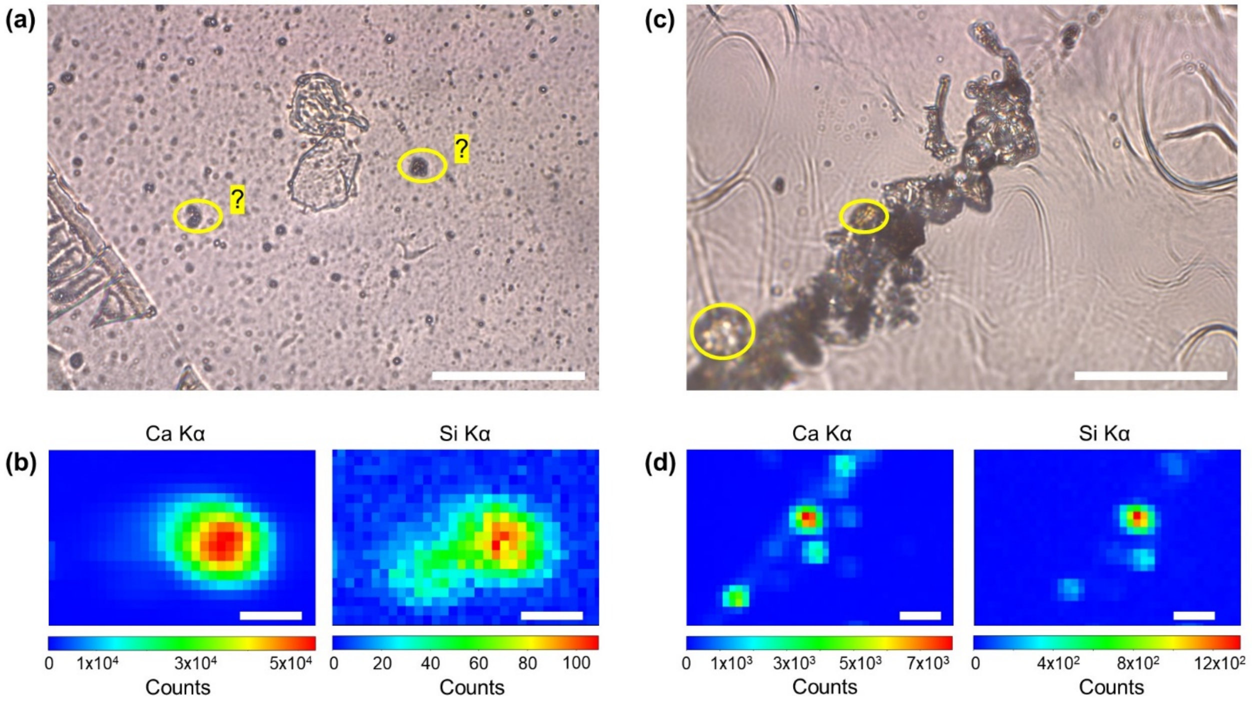

Figure S1. Data collected at XRF beamline on fossil coccoliths of *H. carteri* from sample F1 (**a-b**) and F2 (**c-d**). Micrographs acquired at light microscope in transmission mode at 400X magnification (**a, c**). Scale bar of 50 μm is also reported. Identifiable coccoliths are highlighted in yellow. XRF maps of Ca Kα and Si Kα lines collected at 5 keV incident beam, highlighting their co-localization (**b, d**). Spatial resolution is different among samples and compared to the cultivated sample due to the larger amount of material available for the cultures compared to the fossil samples. Spatial resolution is equal to 20x20 μm^2, scale bar 150 μm (b), and 50x50 μm^2, scale bar 300 μm (d). Counts are normalized to the incident I0 intensity. Maps were generated with the PyMCA software package[36] (https://pymca.sourceforge.net/).

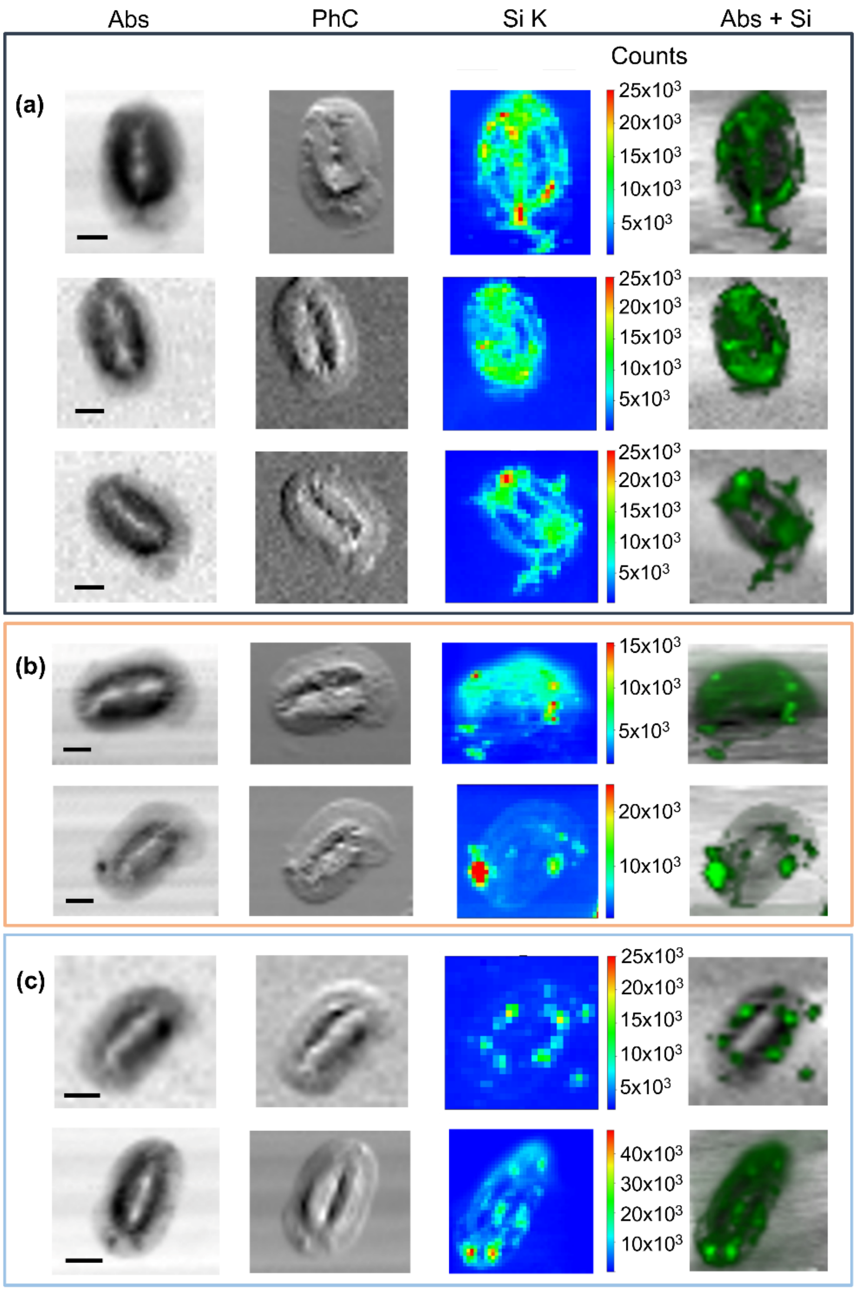

Figure S2. Additional coccolith XRF maps collected at TwinMic beamline on coccoliths from culture sample C1 (**a**), and fossil samples F1 (**b**) and F2 (**c**). Absorption (Abs) and phase contrast (PhC) images of coccoliths are depicted together with the corresponding Si XRF map (Si_K) and the overlapping image of absorption and Si distribution (Abs + Si). All images were acquired at 1.92 keV with 300 nm step size and 60ms acquisition time for Abs and PhC, while 15s for XRF map. Scale bar is 2 µm. Color bars report the intensity counts. Maps were generated with the PyMCA software package[36] (https://pymca.sourceforge.net/).

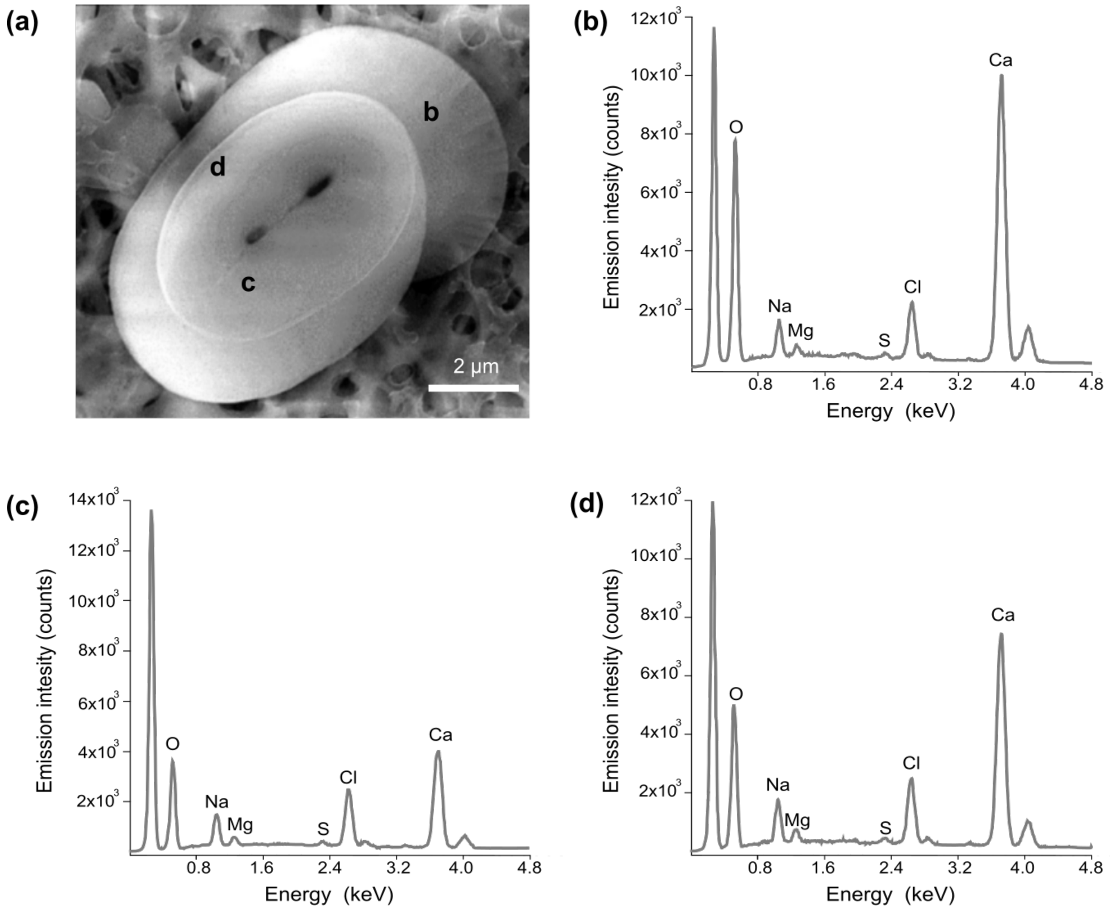

Figure S3. Energy Dispersive Spectroscopy (EDS) measurements on very well-preserved cultured coccolith of *H. carteri* isolated from sample C1. **a**) Secondary Electron SEM image of *H. carteri* single coccolith collected at 25,000 X magnification; **b-c-d**) EDS spectra acquired on different sectors (b-c-d) of the photographed *H. carteri* coccolith. The detected elements are also reported: O (oxygen), Na (sodium), Mg (magnesium), Cl (clorum), and Ca (calcium). All the EDS measurements were processed with EDAX Spectrum Viewer software (https://www.edax.com/support/spectrum-viewer).
